# Capacity, Financing, and Structural Strain in Mental Health Care in the United States: A Narrative Review

**DOI:** 10.7759/cureus.100635

**Published:** 2026-01-02

**Authors:** Abdulazeez Alabi, Olamiji T Onafowokan, Tope Amusa, Olajide Akinpeloye, Deborah Okunola

**Affiliations:** 1 Mathematics and Statistics (Biostatistics), Georgia State University, Atlanta, USA; 2 Biostatistics, Georgia State University, Atlanta, USA; 3 Epidemiology and Medical Statistics, University of Ibadan, Ibadan, NGA

**Keywords:** behavioral health financing, psychiatric bed capacity, structural strain, system rebalancing, workforce shortages

## Abstract

The strain on the US mental health care system is structural, evidenced by a high demand for services that outstrips partial treatment coverage and limited behavioral health spending. This deficit has widespread consequences, spilling over and burdening emergency services, correctional facilities, and homelessness support systems. The narrative review examined interactions among capacity, financing, and cross-sector consequences by synthesizing surveys, claims, workforce projections, facility and crisis-service inventories, and homelessness and justice assessments identified through database and targeted searches. Findings show a high prevalence of mental illness alongside treatment spending that remains a small proportion of national health expenditure. Medicaid finances a disproportionate share of mental health care, and workforce shortages, psychiatric bed deficits, and uneven community infrastructure restrict access, particularly in rural and safety-net settings. The expansion of crisis lines, mobile crisis teams, and Certified Community Behavioral Health Clinics has broadened their reach, but it has not closed gaps in coverage or long-term support. Structural strain concentrated harm in racially minoritized, low-income, and unstably housed populations. System rebalancing through aligned financing reform, workforce retention, and coordinated planning across health, housing, and justice sectors offers a path toward more timely care.

## Introduction and background

Structural strain in the United States' mental health care operates within a context of high and stable population-level need. Recent estimates from national surveys indicate that more than one in five adults experience a mental illness in a given year, and more than one in 20 individuals experience serious mental illness [1,2]. Structural strain refers to systematic misalignment between the need for care, available service capacity, financing arrangements, and the distribution of risk across health, social, and carceral systems. Evidence from emergency departments, jails, and homelessness programs shows repeated diversion of untreated serious mental illness and co-occurring substance use disorders into settings poorly designed for recovery-oriented care [3,4].

Multiple bodies of work describe constrained workforce and facility capacity, underinvestment in community services, and fragmented behavioral health financing. Workforce reports from the Health Resources and Services Administration identify well over 122 million residents living in mental health professional shortage areas, with particularly sparse supply in rural and safety-net settings [5,6]. Reports from the Treatment Advocacy Center and collaborating investigators document steep long-run reductions in state psychiatric beds per capita and widespread operation of remaining facilities with occupancy levels associated with frequent boarding in emergency departments [3,4,7]. Expenditure projections for treatment of mental and substance use disorders show growth that lags overall health spending and stabilizes at mid-single-digit proportions of national health expenditure, reinforcing a gap between burden and investment [8,9]. Parallel evidence from homelessness assessments and cross-sector reviews highlights rising numbers of people experiencing homelessness and disproportionate exposure to serious mental illness and substance use disorders in carceral and housing systems [10]. Previous studies often analyzed singular components, such as bed availability, workforce allocation, or payer composition, in isolation, rather than considering structural strain as a system-wide configuration encompassing health, social services, and the criminal-legal sectors [3,5,8,9].

This narrative review consolidates national and multi-state evidence on capacity, financing, and cross-sector consequences in the United States' mental health care, with emphasis on the period from the late 2000s through early 2025. The review assembles data from national surveys, administrative claims, workforce projections, facility and crisis-service inventories, and homelessness and carceral assessments, prioritizing peer-reviewed sources and authoritative governmental or professional reports. 

The review pursues three objectives. First, the review characterizes the burden-capacity-financing mismatch at the national scale. Second, the analysis clarifies mechanisms through which workforce distribution, bed supply, and financing architecture generate structural strain across health, carceral, and housing systems. Third, the synthesis identifies operational and policy levers that offer feasible pathways toward system rebalancing and reduced cross-sector harm. 

Findings aim to inform clinicians, health-system leaders, public officials, and community advocates seeking alignment between finite resources and population need while reducing reliance on emergency departments, jails, and shelters as de facto components of the mental health system.

## Review

Methods

Study Design

The article adopted a narrative review approach focused on capacity, financing, and cross-sector consequences in the United States' mental health care. The approach prioritized breadth of policy-relevant evidence over exhaustive enumeration of every primary study and did not follow PRISMA or meta-analytic procedures.

Data Sources and Search Strategy

Evidence was obtained through iterative searches of PubMed/MEDLINE, PsycINFO, Web of Science, and Scopus, supplemented by targeted searches of Google Scholar and the websites of federal and professional agencies, including national mental health, behavioral health, workforce, housing, and justice statistics portals. Searches initially covered January 2008 to December 2024, with focused updates through December 1, 2025, to capture recent federal reports and major policy evaluations. Concept-level keyword combinations addressed United States mental health services, psychiatric bed capacity, behavioral health financing, Medicaid, workforce, crisis services, homelessness, and criminal-legal involvement. Filters limited results to English-language, human-focused reports.

Selection and Data Extraction

Eligible sources included peer-reviewed articles, national or multi-state administrative analyses, workforce projections, and official government or professional reports reporting quantitative estimates for prevalence, treatment coverage, financing shares, workforce or facility capacity, or cross-sector outcomes. Editorials, commentaries without new data, single-site quality-improvement reports, and studies restricted to non-United States settings were excluded unless required to clarify definitions. For each included source, study design, population, timeframe, and headline quantitative estimates were extracted and aligned under thematic domains (burden and coverage, workforce, infrastructure, financing, and cross-sector consequences). A narrative synthesis was conducted; no systematic review procedures or meta-analytic techniques were applied.

Results

Evidence from national surveys, administrative data sets, and workforce projections indicates an increasing disparity between the mental health needs of the population, the available capacity, and the financing structures in the United States. Table 1 consolidates key data sources and headline estimates supporting each thematic strand in this section.

**Table 1:** Summary of key national indicators of structural strain in United States mental health care The disease burden, workforce distribution, financing architecture, and cross-sector consequences are all described in this table using headline quantitative estimates and primary data sources. Important conclusions highlight the mismatch between high prevalence and need and inadequate service capacity or inconsistent funding sources. NSDUH: National Survey on Drug Use and Health; NHA: National Health Accounts; CCBHC: Certified Community Behavioral Health Clinics; ED: emergency department; NHAMCS: National Hospital Ambulatory Medical Care Survey

Domain	Study (First Author, Year)	Key Finding	Population/Timeframe
Burden and coverage	Olfson et al. (2019) [11]	~50% treatment coverage for any mental illness; ~67% for serious mental illness; gaps persist with severity	US adults, recent NSDUH cycles
Workforce and rural access	Fortney et al. (2010) [12]	Rural adults with depression less likely to receive/complete psychotherapy (≥8 visits) than urban counterparts	US adults with depression, survey data
Workforce and rural access	Lambert et al. (1999) [13]	Rural Medicaid beneficiaries with depression had fewer ambulatory mental health visits than urban counterparts	Maine Medicaid enrollees with depression
Financing	Mark et al. (2014) [9]	Mental/substance use disorder spending projected at 6.5% of total health spending by 2020 (slower growth)	US national projections, 2010-2020
Financing	Mark et al. (2003) [14]	9.3-13% of Medicaid dollars for mental health/substance use (1984-1997)	US Medicaid, claims/NHA data
Financing and policy	Grogan et al. (2016) [15]	State variations in Medicaid coverage for substance use treatment/opioid meds; carve-outs common	US states, Medicaid surveys
Facility coverage	Mauri et al. (2024) [16]	55.7 of % US population/39.4% of counties in CCBHC areas; rural gaps persist	US, June 2024 geospatial analysis
Cross-sector strain	Nolan et al. (2015) [17]	21.5% psychiatric ED visits resulted in boarding (vs 11% non-psych); longer stays	US EDs, 2008 NHAMCS

Burden and Treatment Coverage

Recent estimates from the National Institute of Mental Health indicate that more than one in five adults, about 59 million people, met criteria for any mental illness in 2022, and roughly 15 million adults (6.0%) experienced serious mental illness [2]. Parallel analyses of the National Survey on Drug Use and Health (NSDUH) reported comparable prevalence and highlighted the highest rates among young adults aged 18-25 years, with any mental illness approaching 30% in that age group in recent survey cycles [18]. In NSDUH analyses, about half of adults with any mental illness received some form of mental health treatment in the previous year, while treatment coverage approached two-thirds among adults with serious mental illness [11].

Aggregate denominators nonetheless point to substantial unmet need. Mental Health America’s analysis of 2019-2020 NSDUH data indicated that 54.7% of U.S. adults with a mental illness, over 28 million individuals, received no treatment. This phenomenon was especially true for young adults and Black and Hispanic populations [19]. Analyses by Olfson et al. show that the probability of receiving treatment rises sharply with impairment severity, but a sizeable minority of adults with serious impairment still receive no mental health care during the year [11]. 

Co-occurring substance use disorders intensify this pattern. Across national surveys and monitoring reports, a consistent signal emerges: a high and rising burden of depressive, anxiety, and substance use disorders, combined with partial and uneven treatment penetration, particularly among younger adults, racially minoritized groups, and people with low income [2,18].

Workforce Capacity and Distribution

The Health Resources and Services Administration (HRSA) estimated in 2023-2024 that approximately 122 million residents lived in federally designated mental health professional shortage areas, and HRSA briefings noted continued increases in designated shortage-area population over the preceding decade [5]. The limited number of mental health professionals, including psychiatrists, psychologists, psychiatric nurse practitioners, and licensed counselors, in relation to the population's needs, is the basis for these designations. This scarcity is particularly acute in rural areas compared to urban centers. Large portions of rural counties report no psychiatrist in residence and depend on itinerant or tele-mental health coverage, while metropolitan areas cluster specialists but still report long wait times for publicly insured or uninsured patients [5].

Workforce projection models released by HRSA anticipate shortfalls across nearly every behavioral health discipline by the mid-2030s, including substantial deficits in psychiatry, addiction medicine, psychology, and social work, particularly in rural and safety-net settings [5]. Burnout and turnover further erode effective capacity. During and after the COVID-19 pandemic, national surveys of behavioral health clinicians indicated that many of them were emotionally drained, morally distressed, and planning to leave their jobs. This was especially true in community mental health centers, where salaries are lower than in private practice and hospital-based roles [20].

Workforce maldistribution also intersects with insurance coverage. The HRSA report and related analyses show that practices relying heavily on commercial insurance often concentrate in affluent suburban markets, whereas Medicaid-dependent and uninsured populations draw primarily on under-resourced community mental health centers and primary care clinics [5]. Claims analyses of Medicaid beneficiaries with depression in Maine found that rural enrollees had fewer ambulatory mental health visits than urban beneficiaries, even after adjustment for supply and patient factors [13]. In another survey data carried out by Fortney et al., rural adults with depression were less likely to receive psychotherapy and, when they did, were less likely to complete a minimally adequate course of eight or more visits than urban adults, with these differences largely mediated by shortages of mental health specialists [12]. These patterns magnify existing disparities in diagnosis, follow-up, and continuity of care for people with serious mental illness and for racially minoritized communities.

Facility and Service Infrastructure

Facility-level capacity trends illustrate a parallel form of structural strain. Historical analyses from the Treatment Advocacy Center documented more than a 75% reduction in state psychiatric hospital beds per 100,000 population between 1970 and the mid-2010s, with most states operating far below commonly cited benchmarks for minimum inpatient capacity [21]. A 2022 survey by the National Association of State Mental Health Program Directors Research Institute reported persistent shortages of adult and child psychiatric beds across nearly all state systems, with bed occupancy often exceeding 85% and many states describing average waits of days to weeks for civil admissions [22]. Growth in general-hospital psychiatric units or residential treatment facilities, particularly for publicly insured patients, has not fully offset these shortages [3, 22].

Community-based infrastructure has expanded in ways that partially counterbalance inpatient contraction but do not fully absorb demand. Certified Community Behavioral Health Clinics (CCBHC) now operate in most states. The National Council for Mental Wellbeing’s 2022 CCBHC Impact data estimated that all 450 active CCBHCs and grantees collectively served about 2.1 million people in 2022 [23,24]. By March 2024, the 2024 CCBHC Impact Report estimated that approximately 3 million clients were being served across 495 CCBHCs in 46 states, the District of Columbia, and Puerto Rico [25]. A 2024 geospatial analysis by Mauri and colleagues estimated that, by June 2024, 55.66% of the US population and 39.43% of counties lay within a CCBHC service area, with coverage concentrated in certain states and markedly weaker in many rural and underserved regions [16,26].

**Figure 1:**
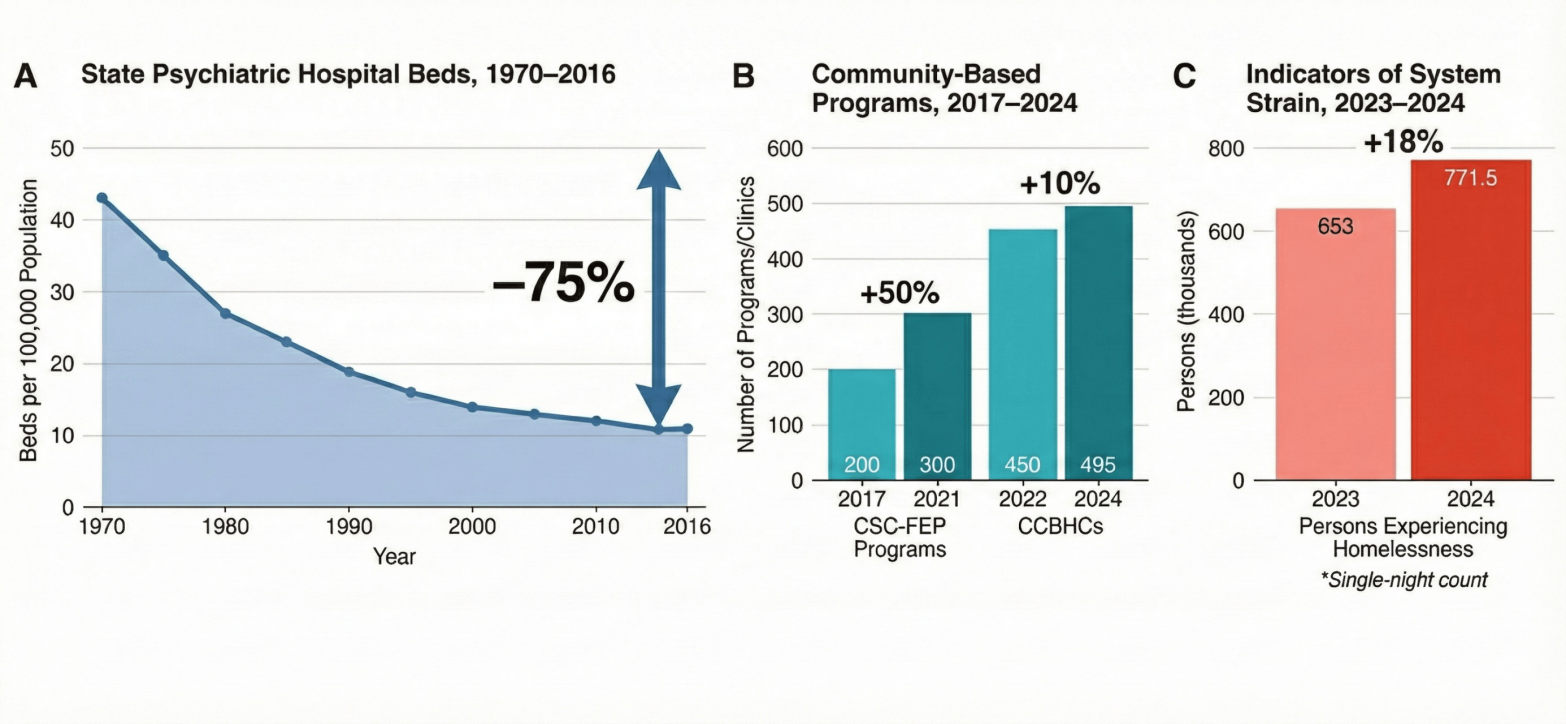
Structural Strain in the U.S. Mental Health Care: Capacity Trends and Cross-Sector Outcomes, 1970–2024. (A) State psychiatric hospital beds per 100,000 population declined by more than 75% between 1970 and 2016. Data approximated from Lutterman et al. (2017) [21], NASMHPD [22], and Treatment Advocacy Center reports [3,4]. (B) Community-based programs, including Coordinated Specialty Care for First-Episode Psychosis (CSC-FEP) and Certified Community Behavioral Health Clinics (CCBHCs), have experienced modest growth in recent years. Data from George et al. (2022) [31], Kazandjian et al. (2022) [33], and the 2024 CCBHC Impact Report [25]. Light bars represent earlier time points; dark bars represent later time points. (C) The number of persons experiencing homelessness (single-night point-in-time count), an indicator of cross-sector strain, increased by 18% between 2023 and 2024. Data from U.S. Department of Housing and Urban Development, 2024 AHAR: Part 1 [10]. Figure created by Abdulazeez Alabi.

National profiling by the National Association of State Mental Health Program Directors Research Institute identified over 544 crisis contact centers for behavioral health, approximately 1,286 mobile crisis teams, and 237 less-than-24-hour crisis receiving and stabilization facilities supported by states in 2022, alongside more than $1 billion in state mental health agency expenditures on crisis services in FY 2022, financed primarily through state general revenue, Medicaid (including 1115 demonstrations), and federal block grants [27-30]. In parallel, SAMHSA and Early Psychosis Intervention Network snapshots show the growth of coordinated specialty care programs for first-episode psychosis from fewer than 200 programs in 2017 to nearly 300 by 2021, with about 24,000 admissions to CSC-FEP services in 2021 across 46 reporting states and 267 programs [31-33]. Figure 1 places these infrastructure trends in a larger context, where small improvements in community capacity happen alongside ongoing problems at the high-acuity end of the system.

Financing Architecture and Spending Patterns

Financing patterns shape both capacity and access. Analyses of national expenditure accounts indicate that direct treatment for mental and substance use disorders accounts for roughly 6-6.5% of total US health care spending, with estimated spending of $186 billion in 2014 (6.4% of all health spending) and projections of $281 billion in 2020 (6.5% of projected national health expenditure) [9,34]. Individuals with mental health disorders nonetheless generate a substantially larger share of overall health expenditures once associated medical spending is counted, and Medicaid alone accounts for about one quarter of all behavioral health spending [34,35]. Behavioral health spending overall reached more than $280 billion in 2020, compared with $172 billion in 2009, against total national health expenditures of $4.5 trillion in 2022 [36-39].

Medicaid has been identified as the single largest payer of behavioral health services in the United States, financing approximately 26% of national behavioral health spending in 2009. National Medicaid claims analyses indicate that about 20% of enrollees carry a behavioral health diagnosis but account for roughly 48% of total Medicaid expenditures, with especially high spending concentrations among disability-based adults and children in child-welfare eligibility categories [35]. Mark et al. suggest that between 9.3% and 13% of all Medicaid dollars were devoted to mental health and substance use services during the 1984-1997 period, based on the most comprehensive claims-based and National Health Accounts studies [14]. Recent payer-mix analyses of behavioral health spending continue to show Medicaid and state and local funds each contributing roughly one quarter of national behavioral health expenditures, with private insurance and Medicare supplying most of the remaining financing [35]. State-level surveys of Medicaid behavioral health coverage describe substantial variation in covered services, use of managed-care carve-outs, and integration of behavioral and physical health benefits across programs [15].

Federal and state initiatives, including expanded financing for Certified Community Behavioral Health Clinics (CCBHCs) and the 988 Suicide and Crisis Lifeline, have channeled new resources into crisis responses and community-based care, as documented in the 2022 CCBHC Impact Report from the National Council for Mental Wellbeing and recent national 988 evaluations by Purtle and Lindsey (2025) [40,41]. The CCBHC survey data and accompanying summaries indicate that caseloads increased by approximately 23% after clinics gained CCBHC status, with an estimated 2.1 million people served across roughly 450 active CCBHCs and grantees in 2022, and about 49% of clinics reporting the addition of new crisis response services or partnerships following certification [41].

Administrative data synthesized by Purtle and Lindsey show that, in the first year after the transition to 988, total lifeline contacts increased by about 40% compared with the prior National Suicide Prevention Lifeline to nearly 5 million calls, texts, and chats, while by March 2024 mean answer times had fallen to roughly 21 seconds and mean in-state answer rates had reached about 85% [41]. Fiscal analyses of 988 implementation by Purtle et al. estimate that fiscal year 2022 state 988 per capita expenditures ranged from about $0.30 to $4.73 (mean $1.15) [42], and policy tracking by the National Alliance on Mental Illness identifies that many states still lack a dedicated 988 telecommunications user fee and continue to rely on limited-term federal and state appropriations, prompting ongoing concern about the sustainability of crisis-system financing [40,43].

Structural Strain Across Crisis, Carceral, and Housing Systems

Structural strain manifests most visibly in settings outside the traditional mental health sector. Emergency departments increasingly function as overflow units for inpatient psychiatry. An analysis of the 2008 National Hospital Ambulatory Medical Care Survey found that approximately 21.5% of psychiatric presentations resulted in boarding, compared to 11% of non-psychiatric visits, and that patients with mental health diagnoses boarded an average of 2.78 hours longer after adjustment [17]. Subsequent state and national surveys describe children and adults with acute psychiatric needs held for days in emergency departments because of the absence of inpatient or step-down options, contributing to crowding, delays in somatic care, and heightened risk for adverse events [22].

Carceral institutions absorb a sizeable share of unmet need. A Bureau of Justice Statistics report based on the 2011-2012 Survey of Prison Inmates estimated that about 43% of state and 23% of federal prisoners had a history of a mental health problem and that roughly 14% of state prisoners met the threshold for serious psychological distress in the past 30 days [44]. A companion analysis of jail inmates reported serious psychological distress in about one quarter of inmates and lifetime diagnoses of a mental disorder in more than 40% [45]. Treatment in these settings often consists of brief medication management in restrictive environments, with limited continuity of care upon release [46].

**Figure 2:**
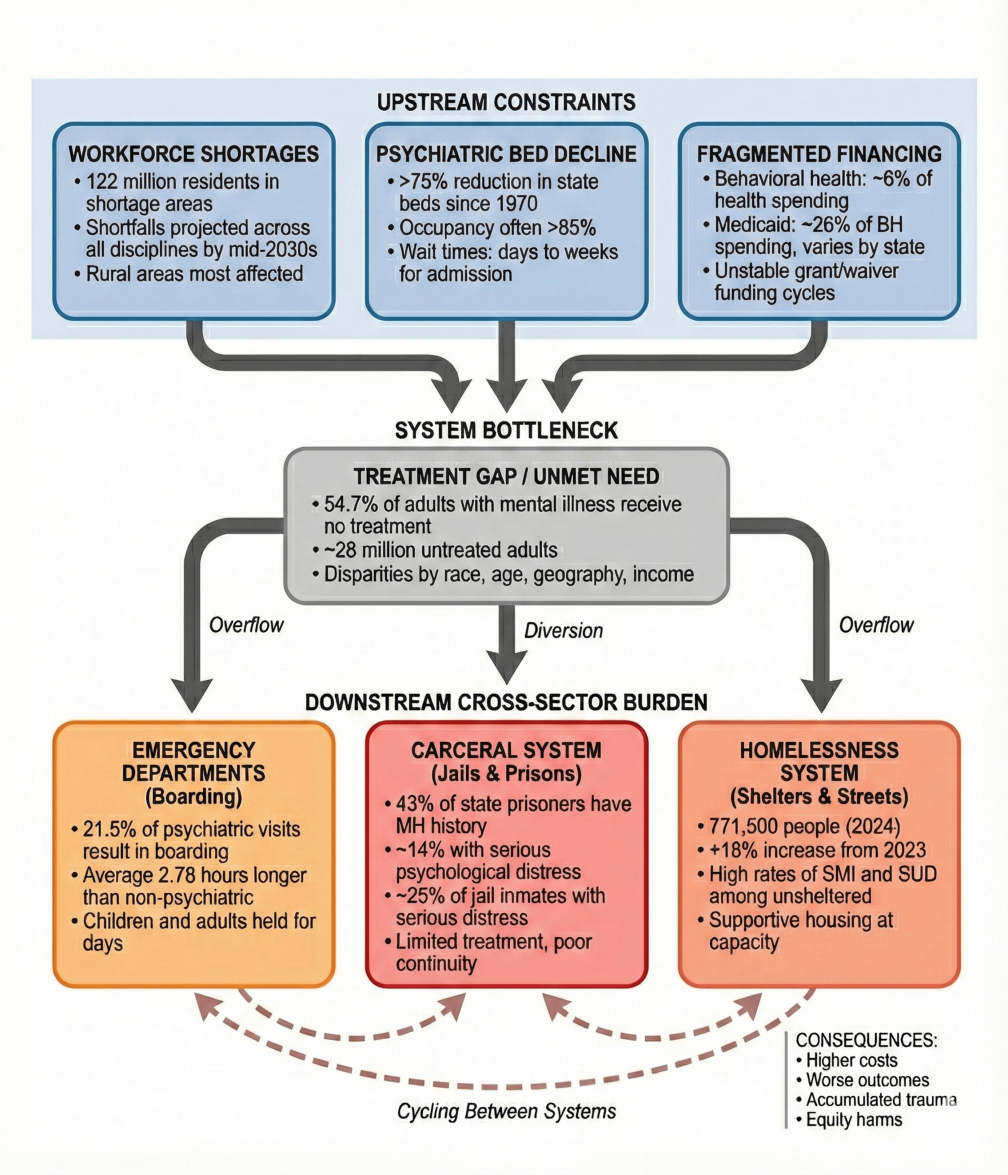
Cross-System Pathways of Structural Strain in U.S. Mental Health Care. Upstream constraints, workforce shortages, psychiatric bed decline, and fragmented financing converge to create a substantial treatment gap, with more than half of adults with mental illness receiving no care [19]. Unmet need propagates downstream into emergency departments, where psychiatric patients experience prolonged boarding [17]; carceral settings, where high proportions of inmates have mental health histories [44, 45]; and homelessness systems, where serious mental illness and substance use disorders are overrepresented [10,48]. Dashed arrows indicate cycling between downstream systems, as individuals with untreated serious mental illness move repeatedly between emergency departments, jails, and shelters without sustained connection to recovery-oriented care. BH: Behavioral health; MH: Mental health; SMI: serious mental illness; SUD: substance use disorder Figure created by Tope Amusa.

Housing systems also show signs of structural strain. The 2023 Annual Homelessness Assessment Report (AHAR) estimated that more than 653,000 people experienced homelessness on a single night in January 2023, representing a 12% increase from 2022 [47]. Early findings from the 2024 AHAR and related summaries reported an additional increase to approximately 771,500 people experiencing homelessness, corresponding to an 18% rise between 2023 and 2024 [10]. Local point-in-time surveys consistently document high rates of serious mental illness and substance use disorders among unsheltered individuals [48]. Community mental health programs and supportive housing providers frequently operate at or near capacity, leaving many individuals with serious mental illness cycling between shelters, street homelessness, jails, and hospital emergency departments [10, 48]. Figure 2 synthesizes these cross-system pathways, depicting how limited upstream capacity and fragmented financing propagate demand into crisis, carceral, and housing settings where care is pricier and less recovery-oriented, and establishing the empirical platform for subsequent analysis of mechanisms, policy trade-offs, and feasible pathways to strengthen capacity and rebalance financing in U.S. mental health care.

Discussion

System-Level Interpretation of Capacity and Financing Patterns

Review of national surveys, expenditure accounts, workforce projections, and service-infrastructure datasets depicts a mental health system stretched beyond design limits. Prevalence estimates for any mental illness and serious mental illness in adults stay in the mid-teens and low-single-digit range, respectively, while treatment coverage holds near roughly half of adults with any mental illness and only modestly higher for serious mental illness, according to recent National Institute of Mental Health statistics and related summaries [1,2]. Co-occurring substance use disorders further amplify morbidity and mortality and increase contact with high-cost settings such as emergency departments, hospitals, and jails, as shown in the National Survey on Drug Use and Health trend data and related federal reports [18].

Behavioral health financing patterns reinforce that mismatch. Pre-COVID expenditure forecasts from Mark et al. suggested that spending on mental and substance use disorders would grow more slowly than total health spending, declining from about 7.4% of all US health expenditure in 2009 ($172 billion of $2.3 trillion) to 6.5% in 2020 ($281 billion of $4.3 trillion) [9]. Medicaid and state and local appropriations finance a large portion of behavioral health care, with the Medicaid and CHIP Payment and Access Commission identifying Medicaid as the single largest payer for behavioral health services and accounting for about 26% of national behavioral health spending in 2009 [35]. Commercial insurance and Medicare supply most of the remaining funding, often with restrictive networks and higher cost-sharing for specialty mental health care. Alignment between high burden and relatively modest, fragmented financing appears weak, particularly for services that reduce downstream cost in other sectors, including supported housing, intensive community treatment, and integrated primary care [49].

Structural strain includes congestion in clinical services and concentrated exposure to coercive encounters, incarceration, and homelessness in specific demographic and socioeconomic groups. Bed shortages, thin community infrastructure, and unstable financing for crisis and housing supports channel individuals with serious mental illness toward emergency departments, jails, and shelters when outpatient treatment or planned inpatient care is unavailable. Evidence describing emergency department boarding, recurrent short-stay hospitalizations, and prolonged waits for competency restoration in forensic settings, therefore, fits a system-level pattern rather than isolated institutional inefficiencies [3,50].

Mechanisms Generating Structural Strain

Structural strain arises when cumulative pressures from burden, financing, workforce limitations, and facility capacity converge on a set of choke points. Psychiatric bed capacity illustrates a central mechanism. Long-run reductions in public psychiatric beds, slow growth in general-hospital psychiatric units, and limited development of subacute and residential alternatives leave many regions with inpatient capacity near or below thresholds associated with frequent boarding. Reports from the American Psychiatric Association and the Treatment Advocacy Center describe widespread operation of state hospitals at or above 85% occupancy, extensive psychiatric boarding in emergency departments, early discharges, and diversion of individuals with serious mental illness into shelters and jails [3,50]. An observational study of boarding in emergency departments reported prolonged stays, delayed somatic care, and higher risks for restraint, seclusion, and adverse events when psychiatric beds are unavailable for extended periods [51]. Bed shortages, therefore, propagate strain into general hospitals, law enforcement agencies, and homelessness systems [3,4,50]. The scarcity of psychiatric beds subsequently extends systemic pressure to general hospitals, law enforcement, and services addressing homelessness.

Workforce dynamics add a second mechanism. The 2024 State of the Behavioral Health Workforce report from the Health Resources and Services Administration, together with earlier HRSA workforce projections, describes substantial anticipated shortfalls across psychiatry, addiction medicine, psychology, social work, counseling, and peer roles, with about 122 million residents living in designated mental health professional shortage areas in 2024 and persistent maldistribution toward metropolitan regions [5,52]. Shortage-area designations cluster in rural and frontier counties, tribal lands, and low-income urban neighborhoods, and state mental health authorities routinely identify workforce scarcity as a leading barrier to service expansion [5]. Even where new funding sources help community programs, the scarcity of qualified staff limits the ability of clinics, crisis teams, and residential programs to expand capacity. High turnover and burnout among behavioral health workers further erode effective supply, especially in safety-net organizations that cannot match salaries offered by hospital systems or private practices. Workforce constraints combine with bed shortages to constrain throughput across the continuum and sustain long waiting lists for intensive community services, such as assertive community treatment, coordinated specialty care for first-episode psychosis, and comprehensive child and adolescent programs [6,20,52].

Financing architecture interacts with workforce and bed constraints in ways that sustain structural strain. Heavy reliance on Medicaid and grant funding for community mental health creates exposure to state budget cycles, waiver approvals, and time-limited federal initiatives. MACPAC analysis shows that beneficiaries with behavioral health diagnoses account for a minority of Medicaid enrollees but a disproportionate share of Medicaid spending, particularly among disability-based adults and children in child-welfare categories, implying high fiscal stakes when states alter behavioral health coverage or payment [35]. Comparative work by Grogan et al. highlights large state-level differences in covered services, managed-care arrangements, and financing details for behavioral health benefits, consistent with the policy heterogeneity described in capacity and crisis infrastructure [15]. States with broad Medicaid eligibility, robust behavioral health benefits, and sustained investment in community infrastructure demonstrate more developed crisis continuums and outpatient networks, whereas jurisdictions with limited Medicaid expansion, narrow behavioral health benefits, or frequent shifts between carve-out and integrated managed-care arrangements report fragmented service arrays and heavier reliance on emergency departments and jails as de facto crisis systems. Grant-funded initiatives such as early waves of Certified Community Behavioral Health Clinic demonstrations and initial 988 implementation dollars can accelerate local innovation, but temporary funding without durable rate structures or statutory telecommunications fees encourages time-limited projects rather than stable system architecture [40,41]. 

Uneven Strain Across Populations and Systems

Patterns of strain do not distribute evenly across populations or geographies. National surveys and administrative data consistently show lower rates of specialty mental health use among Black, Hispanic, and Indigenous populations compared with White populations, even after adjustment for insurance coverage and income, and lower rates of minimally adequate care among racially minoritized groups who do enter treatment [2,15]. Youth and young adults exhibit particularly high prevalence of depressive and anxiety symptoms with relatively low rates of guideline-concordant treatment, as reflected in NSDUH and NIMH age-stratified estimates [2]. Rural residents experience higher travel burdens, limited provider choice, and fewer crisis options, consistent with HRSA workforce and shortage-area distribution patterns [5]. Structural racism, stigma, immigration-enforcement fears, and language barriers further reduce service uptake for many communities [53]. The resulting gradient leaves racially minoritized, rural, and low-income communities with the greatest burden and the thinnest safety nets [54].

Cross-sector consequences follow predictable paths under such conditions. Emergency departments function as overflow units for psychiatric care when outpatient slots, crisis-stabilization beds, and inpatient units cannot absorb demand. Literature on psychiatric boarding describes extended stays, frequent use of security staff, and strained relationships between emergency physicians and community mental health providers [3]. Carceral institutions inherit a large share of untreated serious mental illness through competency-restoration backlogs, diversion gaps, and behavioral symptoms misinterpreted as criminal conduct. National jail and prison assessments report a high prevalence of serious psychological distress and suicidality among incarcerated populations, alongside limited access to evidence-based treatment and disrupted continuity of care [50,55]. Homelessness systems absorb individuals discharged from hospitals and jails who cannot secure stable housing and supportive services, perpetuating cycles of crisis contact, incarceration, and acute hospitalization [10,48].

Equity considerations converge with cross-sector flows. Racially minoritized populations, people with disabilities, and individuals experiencing homelessness face higher rates of coercive encounters with police and security staff, greater exposure to use-of-force incidents, and lower rates of successful linkage to voluntary outpatient care after crisis events. People with serious mental illness and co-occurring substance use disorders who cycle between street homelessness, shelters, emergency departments, and jails accumulate trauma and functional impairment that complicate later engagement in planned treatment. Structural strain, therefore, includes both congestion in clinical services and unequal distribution of criminal-legal and housing harms across demographic and socioeconomic groups [2,10,44,45]. 

Strengths, Gaps, and Contribution to Existing Literature

The evidentiary foundation for this synthesis combines strengths and gaps. National expenditure accounts, Medicaid claims analyses, workforce projections, and bed-capacity inventories provide consistent quantitative anchors and support reliable inference regarding broad trends in financing, workforce supply, and inpatient capacity [3,5,9,35]. In contrast, many studies describing crisis services, jail-diversion programs, coordinated specialty care, and supportive housing rely on cross-sectional designs, single-system case studies, or early implementation evaluations. Such designs clarify where coverage, capacity, and equity gaps occur but provide little leverage to attribute changes to particular policies or payment arrangements. Equity effects often receive cursory attention or appear in subgroup analyses without adequate statistical power, resulting in a partial understanding of how reforms affect different populations [15,40].

Earlier reviews frequently focused on individual domains such as psychiatric bed capacity, behavioral health workforce, or mental health financing. Bed-capacity reviews highlighted long-run institutional downsizing and associated emergency-department boarding and incarceration [3,51]. Workforce reports catalogued shortages and maldistribution without systematically linking the described patterns to funding streams or cross-sector outcomes [5,52]. Financing analyses traced payer mixes and spending growth but devoted less attention to structural consequences in emergency departments, jails, and homelessness systems [9,56]. Alignment of quantitative evidence on capacity, financing, and cross-sector outcomes shows how modest behavioral health spending, Medicaid-dominated financing, workforce scarcity, and limited inpatient capacity interact to generate persistent structural strain despite growth in selected community-based and crisis-response initiatives [3,9,35,52].

Actionable Directions for Financing, Workforce, and System Design

The observed trends across financing, workforce availability, and cross-sector results highlight specific operational and policy areas for intervention. Financing reforms that maintain behavioral health spending at single-digit shares of total health expenditure while concentrating growth in higher-priced inpatient and pharmaceutical services are unlikely to relieve pressure on emergency departments, jails, and homelessness systems. Payment models for behavioral health that reward long-term community involvement, stable housing, and fewer crisis contacts would more directly address the mechanisms found in the synthesis. A workforce policy that couples training-expansion initiatives with retention strategies, loan-repayment programs, and investment in supportive work environments would address effective supply rather than nominal headcounts alone. Capacity-planning processes that link psychiatric bed targets, crisis-continuum design, and supportive-housing pipelines to jail and homelessness flows would create more coherent and resilient systems [5,9,35,50].

Equity-oriented monitoring represents a final cross-cutting implication. Routine reporting of access, quality, coercive contacts, and cross-sector outcomes by race, ethnicity, geography, disability, and housing status would enable earlier detection of disproportionate strain and provide feedback for targeted interventions. Without such measurement, structural strain may appear as a generic capacity problem rather than a pattern that concentrates harm in communities already affected by marginalization. Detailed operational levers, including specific Medicaid waiver authorities, telecommunications fee models for 988, and cross-agency governance structures, belong in the Implications section; the central contribution of this Discussion lies in clarifying key mechanisms that any such reforms must address.

## Conclusions

Structural strain in United States mental health care reflects a persistent configuration of high burden, partial treatment coverage, single-digit behavioral health spending shares, Medicaid-dependent financing, workforce shortages, and gaps in psychiatric and community capacity. Combined effects of those pressures divert serious mental illness and co-occurring substance use toward emergency departments, jails, and homelessness systems, even after substantial investment in crisis lines, community clinics, and specialty programs.

A more credible trajectory centers on rebalancing rather than broad, unspecific expansion. Payment arrangements that reward community tenure, housing stability, and fewer crisis contacts can redirect resources toward assertive community treatment, coordinated specialty care, and supportive housing. Workforce policy focused on retention incentives, supervision quality, and safer practice environments can transform funded positions into durable clinical capacity. Capacity planning that aligns psychiatric beds, crisis infrastructure, diversion pathways, and housing pipelines under routine equity-oriented monitoring of access, coercive encounters, and outcomes offers a practical route for relieving structural strain while strengthening safety and continuity of care.
